# Health-related quality of life and economic impact of urinary incontinence due to detrusor overactivity associated with a neurologic condition: a systematic review

**DOI:** 10.1186/1477-7525-11-13

**Published:** 2013-01-31

**Authors:** Crisanta I Tapia, Kristin Khalaf, Karina Berenson, Denise Globe, Michael Chancellor, Lesley K Carr

**Affiliations:** 1 Health Economics and Outcomes Research, Covance Market Access Services, Inc., 10300 Camput Point Dr, Suite 225, 92121-1511, San Diego, CA, USA; 2 Global Health Outcomes Strategy and Research, Allergan, 2525 Dupont Dr, 92612, Irvine, CA, USA; 3 Health Economics and Outcomes Research, Covance Market Access Services, Inc., 9801 Washingtonian Blvd, 9th Floor, 20878, Gaithersburg, MD, USA; 4 Department of Neurourology, William Beaumont Hospital, 3601 West 13 Mile Rd, 48073-6769, Royal Oak, MI, USA; 5 Division of Urology, Sunnybrook Health Sciences Centre, 2075 Bayview Ave, Unit MG501, M4N 3M5, Toronto, ON, Canada

**Keywords:** Urinary incontinence, Overactive bladder, Quality of life, Burden of illness, Health resources, Economic burden

## Abstract

**Background:**

Patients with neurologic diseases often have neurogenic detrusor overactivity (NDO), which can result in a loss of voluntary bladder control and uncontrollable urinary incontinence (UI).The impact of UI due to NDO on patients’ lives has not been well studied. The objective of this review was to assess the health-related quality of life (HRQoL) and economic burden in patients with urgency UI due to NDO in select countries in North America, the European Union, Asia, and Australia.

**Methods:**

Systematic literature searches and reviews of articles published in English (January 2000 to February 2011) were conducted using MEDLINE®, EMBASE®, and the Cochrane Library. Studies assessing the impact of UI on HRQoL of patients with an underlying neurologic condition of interest (i.e., multiple sclerosis, spinal cord injury, Parkinson’s disease, stroke, or spina bifida) were included. Economic studies in urgency UI also were included.

**Results:**

Of 876 citations generated in the initial search, a total of 27 articles were deemed relevant: 16 articles presented HRQoL data and 11 articles presented information on the economic burden of UI. Humanistic studies used a range of HRQoL instruments to measure HRQoL burden, and the economic studies included different cost components to quantify the economic burden, making meaningful comparisons challenging. Despite this heterogeneity, the literature suggests that HRQoL in patients with UI due to NDO is worse than patients with UI in general or those with the same underlying neurologic condition without UI. In addition, urgency UI also results in substantial economic costs.

**Conclusions:**

Incontinent patients with underlying neurologic conditions have impaired HRQoL as well as substantial economic burden attributable to UI due to NDO. There is a need for urgency UI treatments that improve HRQoL of these patients and alleviate the economic burden of this condition.

## Review

### Background

Patients with neurologic diseases such as multiple sclerosis (MS) or spinal cord injury (SCI) often have a loss of bladder control. This may result from involuntarybladder contractions during the filling and storage phase of the urination process, which is referred to as neurogenic detrusor overactivity (NDO) [1,2]. Symptoms of NDO mirror those of overactive bladder (OAB), and include urgency, urinary frequency, and urgency urinary incontinence (UI), and may lead to serious medical sequelae such as urinary tract infections and skin decubiti if inadequately managed [3-8].

Although numerous studies have quantified both the economic and quality-of-life impact of OAB and general incontinence symptoms, the impact of UI specifically due to NDO has not been well characterized. Due to their underlying neurologic conditions, patients with UI and NDO may view their urinary symptoms differently than their otherwise healthy counterparts, and urgency UI due to NDO is secondary to numerous neurologic conditions that vary not only with respect to their presentation but also in the characteristics of affected patients. A better understanding of the negative effects of urinary symptoms on the economic outcomes and quality of life of patients with neurologic conditions is important in order to optimize treatment when potential pharmacologic therapies may be appropriate, as the utilization of outcomes data together with clinical assessments best reflects the patients spectrum of disease [1]. A systematic review of the published literature was therefore conducted in order to describe the health-related quality of life (HRQoL) and estimate the per-patient economic burdens of urgency incontinence due to NDO secondary to any of the following neurologic disorders: MS, SCI, Parkinson’s disease, stroke, or spina bifida.

## Methods

### Study design

Literature searches were conducted using MEDLINE®, EMBASE®, and the Cochrane Library, which included Cochrane Reviews, the Database of Abstracts of Reviews of Effects (DARE), the Cochrane Central Register of Controlled Trials (CENTRAL), the Health Technology Assessment Database, and the National Institute for Health Research Economic Evaluation Database (NHS EED), to identify relevant articles that assessed the humanistic and economic burden of UI due to NDO. Relevant search terms for UI, such as incontinence, neurogenic bladder, detrusor overactivity, and urgency, were cross-referenced with outcome concepts (HRQoL burden of illness and economic burden of illness) as appropriate and with terms for the following underlying neurologic conditions: MS, SCI, Parkinson’s disease, stroke, and spina bifida. The search was limited to titles and abstracts of human studies conducted in the United States, the European Union 5 (EU 5: United Kingdom, France, Germany, Italy, and Spain), Canada, Australia, Thailand, South Korea, and Taiwan that were published in English from January 2000 to February 2011. Selected studies were ranked and assigned levels of evidence in accordance with the 2011 Oxford Centre for Evidence-Based Medicine (CEBM) Levels of Evidence [9].

### Study selection criteria

The search failed to identify burden of illness studies in individuals with urodynamically confirmed NDO. Since the literature suggests that UI among patients with underlying neurologic conditions is attributable to detrusor overactivity in the majority of cases, [10,11] the inclusion criteria were slightly modified to include any burden of illness studies in individuals with UI who had one of the underlying neurologic conditions of interest.

In addition, the search identified only one interventional study that assessed the economic burden of UI specifically resulting from NDO.assessed the economic burden specifically resulting from Therefore, these inclusion criteria were modified to include any economic studies that assessed the economic burden of UI in general and reported per-patient costs of care, as it was determined that costs associated with leakage of urine, irrespective of the underlying cause, would not differ substantially by UI etiology. Recognizing that the UI population is much larger and distinct from the NDO-related UI population, only studies that presented per-patient costs (vs. total costs for the UI population) were ultimately included to conservatively assess the economic impact of UI on patients with NDO.

### Literature selection and data extraction

The selection and appraisal of the titles, abstracts, and literature were completed by two independent reviewers. The reviewers documented the reason for exclusion for every article that was not included in the analysis. Any discrepancies in the selection and appraisal of the articles were discussed between the reviewers and resolved with the help of a third reviewer, where necessary. Full articles were retrieved for the remaining documents from this round of review. The inclusion/exclusion criteria were applied to the review of full articles and key relevant articles were selected for data extraction. The key data, including a description of the study design, study population, baseline characteristics, and a summary of the economic and humanistic burden measures and related findings, were extracted from each publication. In order to compare costs related to the per-patient economic burden of UI reported across studies from different years and in various countries, all non-acute costs were annualized by multiplying weekly costs by 52 and daily costs by 365, and all costs were converted to US dollars for the year 2010, using consumer price indices for medical resources [12-14].

## Results

**Figure 1:**
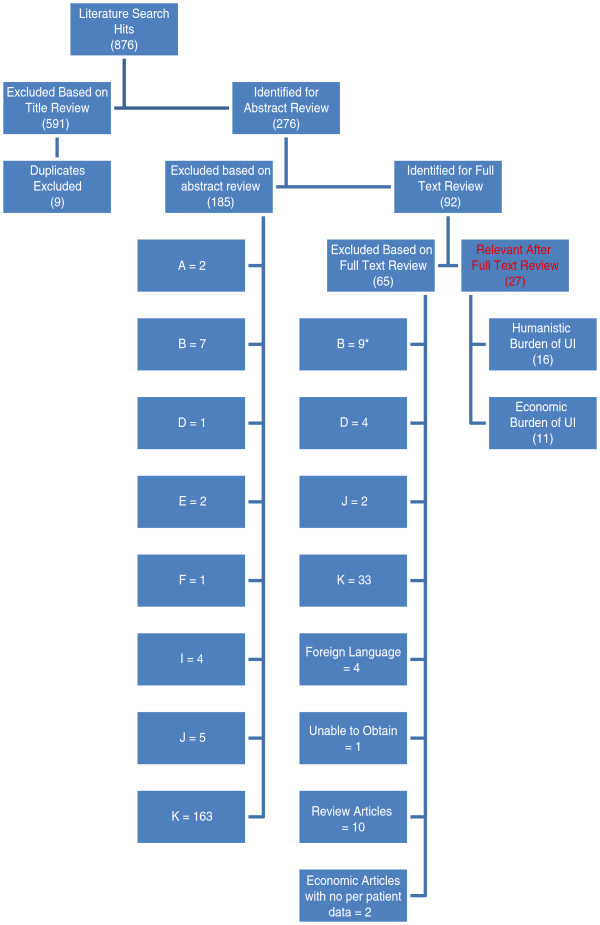
**Article selection flow chart for UI due to NDO publications.** Reasons for exclusion: **A**. Editorials, letters, case reports, lectures, news, comment, legal cases, newspaper article, technical report, and clinical trial studies. **B**. Studies on urinary incontinence (UI) due to idiopathic detrusor overactivity or idiopathic overactive bladder.* **C** Studies in patients with neurogenic detrusor overactivity (NDO) with urgency but without UI (DRY). **D**. Studies on stress UI. **E**. Studies in patients with NDO that is not associated with multiple sclerosis (MS), spinal cord injury (SCI), Parkinson’s disease (PD), stroke, or spina bifida (SB). **F**. Studies in children or adolescents (<18 years of age) with MS, SCI, PD, or stroke. **G**. Studies in patients with overactive bladder (OAB) that do not report results specific to urgency UI. **H**. Studies not requiring patients to have urgency. **I**. Small (n < 30) case series. **J**. Studies from countries other than the US, EU 5 (UK, France, Germany, Italy and Spain), Canada, Australia, Thailand, South Korea, and Taiwan. **K**. Studies that do not specifically discuss the economic or humanistic burden of UI due to NDO from MS, SCI, PD, stroke, or SB. Note: Studies in children or adolescents (<18 years of age) diagnosed with SB were included in the systematic review. * Did not apply to economic studies.

The initial literature search generated 876 citations (Figure 1). After title review, 591 articles were excluded; the majority of these articles were clinical trials evaluating various treatments, which focused primarily on clinical endpoints, rather than HRQoL or economic burden. Abstracts of the remaining articles were then review, after which 92 articles were considered relevant and 87 full-text articles were retrieved (four articles were not available in English and one article was not available from the library or electronic source). Of 87 references, 60 were further excluded because they were not for the population of interest or did not discuss the humanistic or economic burden of UI. Figure 1 presents the article selection criteria for the publications identified from the MEDLINE®, EMBASE®, and the Cochrane Library databases, along with details regarding exclusion criteria.

A total of 27 full text articles fulfilled all of the revised criteria and are summarized in this literature review. No studies assessing the HRQoL burden in patients with UI due to urodynamically confirmed NDO were identified. Sixteen references on the impact of UI on HRQoL in individuals with MS, SCI, Parkinson’s disease, stroke, or spina bifida were identified (Additional file 1: Table S1). As mentioned above, our initial search identified only one study in patients with SCI, spina bifida or MS on the per-patient economic burden of UI in patients with NDO [15]. Our modified search identified 10 additional studies that presented patient-level information surrounding the economic burden of UI in general (Table 1).

**Table 1:** Studies on the economic burden associated with incontinence

**Citation; CEBM Evidence grade **[9]	**Study design**/**methodology**	**Cost data source**	**Country**	**Populations**
Frantz *et al*. 2003 [39]; 2	Secondary data analysis	Medical records	US	Residents of long-term care facility
				• N = 63
				• Mean age 87 ± 7 yr
				• 77% were female
				• 54% were incontinent
Green *et al*. 2003 [34]; 3	Retrospective analysis	Registry	Australia	6773 episodes of care provided to 6455 rehabilitation and geriatric evaluation and management patients
			New Zealand	
Irwin *et al*. 2008 [36]; 3	Economic cost of illness model; included costs for 2002 ICS-defined OAB and urgency UI-related comorbidities and nursing home costs	Economic model	Canada	EPIC survey population with OAB or urgent UI
			Germany	
			Italy	• Aged 18+ yr
			Spain	
			Sweden	
			UK	
Morris *et al*. 2005 [33]; 3	Prospective burden of illness study	Prospective chart data	Australia	Incontinent patients
				• Mean age 85 (range 74–91) yr
Papanicolaou *et al*. 2005 [35]; 3	Prospective urinary incontinence research (PURE) is a non-interventional, longitudinal, prospective, observational, multicentre, multinational study to determine the direct cost for patients with UI	Prospective survey data	Germany	Patients with UI
			Spain	• Germany: n = 2696, mean age 65.1 ± 13.4 yr
			UK/Ireland	
				• Spain: n = 2127, mean age 57.9 ± 12.7 yr
				• UK/Ireland: n = 1070, mean age 60.1 ± 14.3 yr
Prasopsanti *et al*. 2007 [41]; 3	Economic model using diagnostic and treatment algorithms from clinical practice guidelines and current disease prevalence data	Economic model	Thailand	Patients with UI (prevalence estimates used in model derived from elsewhere)
Shih *et al*. 2003 [40]; 3	Prospective study	Prospective survey data	US	Patients with UI in long-term care facilities
Subak *et al*. 2006 [42]; 3	Cross-sectional study	Prospective survey data	US	Women with UI
				• N = 293
				• Mean age 56 ±11 yr
Tediosi *et al*. 2000 [37]; 3	Cross-sectional study/home interview with costs quantified for stress UI and other types of UI separately	Interview data and retrospective database (Italian National Health Service tariffs)	Italy	Women with UI
				• N = 229
				Age >40 yr
Thom *et al*. 2005 [38]; 3	Database study	Retrospective database	US	Women with UI
				• N = N/A (combination of large public and private population data sources, including CMS, MEPS, NHANES, NCHS, VA, NACHRI, and MarketScan)
				• Age >60 yr
Wefer *et al*. 2010 [15]; 2	Multicentre, cross-sectional, retrospective cohort study	Retrospective chart review	Germany	Neurological disorders:
				SCI, 80.8%
				MS, 5.2%
				Spina bifida, 14.0%
				• N = 214
				• Mean age 38 (14.8) yr

*UI* = urinary incontinence; *OAB* = overactive bladder; *CEBM* = Oxford Centre for Evidence-Based Medicine; *CMS* = Centers for Medicare and Medicaid Services; *MEPS* = Medical Expenditure Panel Survey; *NHANES* = National Health and Nutrition Examination Survey; *VA* = Department of Veterans Affairs; *NACHRI* = National Association of Children’s Hospitals and Related Institutions.

### Impact of UI on the HRQoL of individuals with underlying neurologic conditions

Studies that assessed the impact of UI resulting from underlying neurologic conditions on the HRQoL of individuals used a variety of generic and/or disease-specific instruments and focused on different domains of HRQoL. However, the studies generally indicated that UI negatively affects HRQoL among individuals with underlying neurologic conditions of interest (Additional file 1: Table S1).

#### Multiple sclerosis

Four studies provided information on the impact of UI in individuals with MS [16-19]. Two of these studies [16,17] assessed general health status as measured by the Medical Outcomes Study 36-Item Short Form Health Survey (SF-36) [20], a subjective measure of health status across eight domains with scores ranging from 0 (worse HRQoL) to 100 for each scale. The conclusions of the two studies differed, with the first study concluding that UI was not associated with SF-36 scores (independent of the impact of MS itself) [16] while the second study found that for the physical functioning and role-emotional domains, those with UI had lower SF-36 scores compared to those without UI (physical functioning: 42.67 vs. 62.42; role-emotional: 27.13 vs. 42.39, respectively; p < 0.05) [17].

The third study assessed the impact of bladder problems on HRQoL in individuals with MS using disease-specific measures, including the Incontinence Impact Questionnaire (IIQ-7), Urogenital Distress Inventory (UDI-6), and American Urological Association (AUA) Symptom Index. [18] Approximately 96% of individuals reported having bladder problems, with 41% reporting they were “moderately” or “greatly” bothered by frequent urination, 39% by UI associated with urgency, and 26% by activity. Furthermore, a substantial number of patients reported “somewhat” or “a great deal of” interference caused by bladder problems, and urinaryu problems impacted emotional health (31%), ability to perform household chores (22%), and physical recreation (28%). In addition, almost half of the patients reported that they would feel “mostly dissatisfied,” “unhappy,” or “terrible” if they had to spend the rest of their lives with their current urinary condition. These results indicate that UI is not only bothersome and troubling to people with MS but also results in substantial disability.

The fourth study compared HRQoL of OAB patients with MS (OAB+MS group) versus OAB patients without MS (OAB group) using select domain scores of the Kings Health Questionnaire (KHQ; another disease-specific HRQoL measure) and found that urinary symptoms had more impact on HRQoL in the OAB+MS group compared with the OAB group [19]. KHQ scores were significantly higher (indicating greater impairment) in the OAB+MS versus the OAB groups (respectively) in five domains: general health perception (56.3 vs. 33.3, p = 0.02), role limitations (50 vs. 36.1, p = 0.03), physical limitations (56.3 vs. 33.3, p = 0.02), social limitations (45.8 vs. 18.5, p = 0.002), and urinary symptoms (17 vs. 14, p = 0.05). Patients in the OAB+MS group, however, felt that their bladder symptoms had a lesser impact on their lives than those in the OAB group and perceived their incontinence symptoms to be less severe compared with the OAB group. Urinary symptoms in both groups were also shown to adversely affect including physical activities, sleep/energy, emotions, and relationships; however, this impairment was generally greater in patients with MS.

#### Spinal cord injury

Four studies were identified assessing the impact of UI among patients with SCI [21-24]. The first study compared SF-36 scores of 132 incontinent SCI patients using clean, intermittent catheterization (CIC) with those of the general population [22]. Incontinent SCI patients had significantly lower SF-36 scores across all domains (except for the energy and vitality scores in female cases) when compared with the general population (p < 0.05). The greatest detriments in incontinent SCI patients were in the physical functioning domain for both male and female patients (male patients vs. controls, 18.4 ± 3.2 vs. 85.3 ± 1.7, respectively, p < 0.001 and female patients vs. controls, 28.3 ± 4.4 vs. 72.0 ± 2.3, respectively, p < 0001). Scores for all other domains were also lower among SCI patients compared with the general population (detriments ranged from 7.7 to 55.6 points).

Similar results were reported in a separate cross-sectional study in 142 SCI patients in the UK [21]. SF-36 scores and KHQ scores of SCI patients with normal voiding function were compared with scores of incontinent SCI patients who were using various methods of bladder management. Incontinent SCI patients scored significantly lower than continent patients on the physical functioning and mental health domains of the SF-36, as well as on the physical component summary (PCS) and mental component summary (MCS) scores (p < 0.05). Moreover, incontinent SCI patients who were using CIC by an attendant, indwelling transurethral catheterization, or indwelling suprapubic catheterization had the lowest mental health domain and MCS scores compared with SCI patients who were continent or SCI patients using other forms of bladder management, such as self-CIC, reflex trigger, or bladder expression. KHQ scores corroborated these findings. SCI patients without UI had higher KHQ scores compared with incontinent patients requiring bladder management, with significant differences observed in the physical limitations, social limitations, personal relationships, and emotions domains (p < 0.05). The frequency of UI also had a substantial impact on HRQoL. Worse HRQoL scores were associated with increased frequency of incontinence, and SCI patients who experienced daily UI episodes had the worst SF-36 and KHQ scores. Mental health domain and MCS scores significantly decreased as the frequency of UI episodes increased (mental health: 77.4, no incontinence vs. 60.7, daily incontinence; p = 0.01; MCS: 42.2, no incontinence vs. 27.7, daily incontinence; p = 0.01). Significantly worse KHQ scores were also observed in the incontinence impact, social limitations, emotions, and severity measures domains as the frequency of UI increased (p < 0.05).

Baseline data from a recent interventional, randomized controlled trial in SCI patients with incontinence due to NDO further suggest that the HRQoL of these patients is greatly impaired [23]. HRQoL was assessed with the Incontinence Quality of Life (I-QOL) questionnaire and SF-36 before treatment with botulinum toxin A. I-QOL is a 22-item disease-specific HRQoL questionnaire with possible scores ranging from 0 (worse HRQoL) to 100 (higher HRQoL). At study baseline, the mean I-QOL score was 44 (range 9–82), with the greatest decrement noted on the social embarrassment domain (mean score = 33), and SF-36 scores ranged from 51 to 65, except for physical functioning, the average of which was 32. TheT authors noted that the physical functioning score might be more reflective of the physical impact of the neurologic condition rather than that of incontinence. However, significant improvements in the role-physical and role-emotional domains of the SF-36 were observed following botulinum toxin A treatment, implying that some degree of their general HRQoL impairment could be attributed to incontinence.

In addition to physical, mental, and psychological impairments, the final study in SCI patients suggests that UI also negatively affects sexual life [24]. Study participants with traumatic SCI reported via questionnaire that UI caused inconvenience with regards to sexual life. Those reporting that UI was more of an inconvenience in their lives were also more dissatisfied with their sexual life compared with those reporting that UI was less of an inconvenience. This correlation was significant for men but not for women (Spearman’s rho = 0.29, p = 0.021 vs. –0.18, p = 0.236, men vs. women, respectively).

#### Parkinson’s disease

Two relevant studies that assessed the impact of UI in people with Parkinson’s disease were identified. One study in Italy used the symptom bother component of the OAB questionnaire Short Form (OAB-Q SF) to assess the frequency and bother of frequency, urgency, nocturia, and incontinence symptoms in patients with Parkinson’s disease compared with healthy controls [25]. Scores ranged from 8 to 48, with higher scores indicating greater symptom bother. The study found that patients with Parkinson’s disease and urinary symptoms were significantly more bothered compared with healthy controls without Parkinson’s disease or urinary symptoms (18.71 vs. 12.37, respectively; p = 0.0001).

A study in Canada compared HRQoL of patients with Parkinson’s disease and UI versus Parkinson’s patients without UI [26]. The study utilized data from the Canadian Community Health Survey in which health status was assessed using the Health Utilities Index Mark 3 (HUI3), a 31-item questionnaire that assesses usual health status with the following eight attributes: vision, hearing, speech, ambulation, dexterity, emotion, cognition and pain, and discomfort. HUI3 scores range from −0.36 (worst possible health) to 1.0 (1.0 = perfect health). Overall utility scores were significantly lower in persons afflicted with both Parkinson’s disease and UI compared with those with Parkinson’s disease who did not have UI (0.39 vs 0.61, respectively, p < 0.05) or the general population (0.39 vs 0.83, respectively, p < 0.05). Furthermore, clinically important and statistically significantly lower scores were reported for the ambulation (−0.22 [CI −0.42 to −0.02, p < 0.05]), and emotion (−0.28 [CI −0.43 to −0.12, p < 0.05]) attributes by respondents with Parkinson’s disease and UI compared with those who were continent. The study thus suggests that UI has detrimental effects on both the physical and mental health of patients with Parkinson’s disease.

#### Stroke

Five studies in stroke patients with UI were identified, which suggest that incontinent stroke patients have impaired functioning, lower life satisfaction, and higher rate of institutionalization compared with stroke patients who are continent.

Edwards *et al*. [27] interviewed 361 individuals 6 months post stroke and evaluated the impact of UI on functioning,life satisfaction, and social participation. In all, 16% of individuals were incontinent at 6 months, and these individuals were more impaired with respect to basic and instrumental Activities of Daily Living (ADL) scales. Incontinent stroke survivors were significantly less independent with respect to basic self-care, functional communication, and cognition compared with continent survivors (p < 0.0001), as measured by the Functional Independent Measure (FIM). Incontinent individuals also experienced significantly lower HRQoL and well being than continent individuals, as assessed by all other measures, and survivors had significantly lower emotional well-being (SF-12 MCS score) and significantly lower levels of life satisfaction (as measured by the Reintegration to Normal Living Index, RNL) compared with continent individuals (p < 0.0001). On average, incontinent individuals retained only 40% of their pre-stroke activities, whereas continent individuals retained 70%. UI in stroke patients was thus associated with greater dependence in basic and instrumental ADL, decreased participation in normal living, and lower life satisfaction. In a cross-sectional, clinical survey conducted in patients at least 1 month after their incident stroke (n = 407) at an acute stroke and neurological unit in Denmark [28], found that activity limitations were closely related to incontinence. Mobile velocity was significantly associated with severity of urinary symptoms (mean effect = 0.37, p = 0.01), and low Barthel Index (a measure of disability status) scores and reduced mobility velocity increased the risk of the prevalence (OR = 2.08, p = 0.03, and OR = 1.87, p = 0.05, respectively) and severity (mean effect = 0.21, p = 0.03 and mean effect = 0.22, p = 0.04, respectively) of incontinence symptoms.

The remaining three studies assessed the impact of UI on long-term outcomes in stroke patients, including disability, rate of institutionalization, and mortality [29-31]. Kolominski-Rabas *et al*. [29] reported that, according to the Barthel Index, patients who were incontinent after a stroke were severely (39%) or moderately (18%) disabled at 2 years, whereas those who were continent after their strokes were only mildly disabled (26%) or independent (51%).

The rate of institutionalization among incontinent stroke patients ranged from 34% at 3 months post stroke [30] to 45% at 12 months post stroke [29], and was found to be significantly higher among incontinent stroke patients institutionalized at 1 year (45%) compared with those who were continent (5%; p = 0.001). [31] The risk of mortality at 2 years was also greater among stroke patients with UI compared with those without UI (odds ratio 4.43; 95% CI: 1.76 to 11.2; p = 0.002) [31].

#### Spina bifida

The literature search identified two studies that assessed the impact of UI on HRQoL in people with spina bifida; however, neither study found a strong association between UI and HRQoL.The study by Valtonen *et al*. [24] (described above, which assessed sexual satisfaction and level of inconvenience due to UI among SCI patients) also assessed the impact of UI on the sexual life of individuals born with meningomyelocele and found that although UI caused some inconvenience, this inconvenience did not affect sexual satisfaction. The only other study that assessed the relationship between UI and HRQoL in spina bifida was a cross-sectional study in 460 patients at six centers in France using the SF-36 and VSP (Vecu et santé percu, a French generic HRQoL instrument) [32]. HRQoL scores in incontinent adults with spina bifida were not significantly different from those patients without UI, except for the bodily pain domain of the SF-36, in which the continent patient group had a higher score (p = 0.05). No significant differences were observed in the VSP scores among adolescent patients with UI compared with those without UI [32].

### Economic burden of UI

Our search identified one article that assessed the economic burden of patients with urodynamically confirmed NDO, and 10 articles that presented per-patient costs of UI in the general population, 2 of which reported results of subgroups of patients with neurologic disease [33,34] (Table 1). These studies were diverse in terms of perspective (payer, provider, patient, society), the type of data used (electronic medical records, surveys, interviews, model), cost components assessed (labor costs, nursing home costs, office visits, surgery, medications, diagnosis, pad usage), country of data origin (US, Australia, Italy, Spain, Germany, Sweden, UK, Thailand, Ireland), and study design (retrospective, cross-sectional, prospective). The heterogeneity of these economic studies did not allow for direct comparisons of findings across patient populations or countries, yet taken together, they suggest that UI results in significant economic burden from the perspective of the payer, provider, patient, and society as a whole.

One study found the total annual per patient cost of UI to be $613.10 in Germany, $779.76 in Spain, and $427.38 in the UK/Ireland when medication, incontinence products, clinic/physician visits, and hospital stays related to surgical interventions for incontinence were included. The highest contribution to the overall cost was pad usage in Germany (51%), diagnostic procedures in Spain (27%), and health care provider visits in the UK/Ireland (27%) [35]. In another cross-sectional, population-based survey conducted in Canada, Germany, Italy, Sweden, and the UK, direct medical cost estimates (US$) for physician office visits and surgery related to UI from the payer perspective ranged from $62.05 (in the UK) to $259.20 (in Spain) per patient per year in Europe (Table 2) [36]. A study that interviewed women in six areas across Italy found that the most burdensome costs associated with UI from the Italian National Health Service was for diapers, which was estimated to be $148.30 annually [37]. These wide ranges can be attributed to the heterogeneity with respect to the different types of medical resources incorporated into the cost estimates for each study and medical resource utilization patterns across different countries.

**Table 2:** **Total costs per patient**, **associated with incontinence in general**

**Citation**	**Country**	**Perspective**	**Time-frame**	**Cost components reported**	**Cost year**	**UI costs**	**Inflated 2010**	**Costs per year (2010)**
							**Total costs**	
				**Labor Costs**				
Green 2003 [34]	Australia, New Zealand	Provider	1 day	Rehabilitation staff	1996	$185.60/day AUD	$267.40/day AUD	*
				Geriatric evaluation staff		$164.62/day AUD	$237.27/day AUD	*
Morris 2005 [33]	Australia	Provider	1 day	Nursing staff supervision	2003	$32.4 AUD	$39.42 AUD	*
Frantz *et al*. 2003 [41]	US	Provider	1 day	Nursing staff supervision	1995	$112	$197.30	$72,014.50
				Nursing assistant time for toileting		$1,361	$2397.56	$5,208.55
						($8.10/day)	($14.27/day)	
Prasopsanti *et al*. 2007 [41]	Thailand	Society	1 yr	Incremental labor costs in LTC	2002	$4.52/shift	$6.15/shift	$6,741.87/yr
						$13.57/day	$18.56/day	
						$4,957/yr	$6,741.87/yr	
Shih *et al*. 2003 [40]	US	Provider	1 yr	Labor costs in LTC	2005	UI = $9.96	Urinary Incontinent = $13.55	UI = $13.55
								Occasionally Incontinent = $11.90
						Occasionally Incontinent = $8.75	Occasionally Incontinent = $11.90	Frequently Incontinent = $14.42
						Frequently Incontinent = $10.60	Frequently Incontinent = $14.42	
				**Diagnosis**				
Papanicolaou 2005 [35]	Germany	Payer	1 yr	Diagnosis	2004	€42	€46.14	$61.27 USD
	Spain			Diagnosis		€186	€216.12	$286.98
	UK/Ireland			Diagnosis		€6	€7	$9.30
Tediosi 2000 [37]	Italy	Payer	1 yr	Diagnosis	1997	L76.142	L100.27	$133.15 USD
				**Routine Incontinence Management (Supplies and Medication)**				
Frantz *et al*. 2003 [39]	US	Provider	6 mo	Barrier cream	1995	$745	$1,312.40	$2,624.80
				Disposable briefs		$723	$1,273.65	$2,547.30
				Bed pads		$304	$535.53	$1,071.06
				Incontinence management		$9.09/day	$16.01	$5,843.65
				Total incontinence management		$1,372	$2,416.94	$4,833.88
Irwin *et al*. 2009 [36]	Canada	Provider	1 yr	Incontinence pad usage	2005	€56	€60.97	$80.96
	Germany			Incontinence pad usage		€66	€71.41	$94.82
	Italy			Incontinence pad usage		€102	€112.12	$148.88
	Spain			Incontinence pad usage		€102	€114.65	$152.24
	Sweden			Incontinence pad usage		€80	€86.26	$114.54
	UK			Incontinence pad usage		€48	€54.95	$72.97
Morris 2005 [33]	Australia	Provider	1 day	Consumables	2003	$6.8 AUD	$8.27 AUD	*
				Total median daily cost for UI		$36.2 AUD	$44.04 AUD	*
Papanicolaou 2005 [35]	Germany	Payer	1 yr	Pad usage	2004	€235	€258.14	$342.78
				Conservative care		€0	€0	$0
				Medications		€107	€117.54	$156.08
	Spain			Pad usage		€235	€273.05	$362.58
				Conservative care		€19	€22.08	$29.32
				Medications		€88	€102.25	$135.78
	UK/Ireland			Pad usage		€55	€64.26	$85.33
				Conservative care		€42	€49.07	$65.16
				Medications		€125	€146.04	$193.93
Prasopsanti *et al*. 2007 [41]	Thailand	Patient	1 wk	Pad usage	2005	$3.7 USD	$4.45 USD	$231.40
Subak *et al*. 2006 [42]	US	Patient	1 wk	Personal care resources	2005	$6.57 USD	$7.90 USD	$410.80 USD
Tediosi 2000 [37]	Italy	Payer	1 yr	Conservative care	1997	L353.523	L465.526	$618.17
				Medications		L25.412	L33.463	$44.44
Wefer *et al*. 2010 [15]	Germany	Payer	1 yr	Urinary reservoir	2006	€0.70/day	€0.74	$358.66
				Pad usage		€1.41/day	€1.50	$727.02
				**Comorbidities**				
Irwin *et al*. 2009 [36]	Canada	Payer	1 yr	Comorbidities	2005	€19	€20.69	$27.47
	Germany			Comorbidities		€21	€22.86	$30.36
	Italy			Comorbidities		€9	€9.80	$13.01
	Spain			Comorbidities		€7	€7.62	$10.12
	Sweden			Comorbidities		€35	€38.11	$50.61
	UK			Comorbidities		€13	€14.15	$18.79
Wefer *et al*. 2010 [15]	Germany	Payer	1 yr	UTI medications	2006	€162.71/yr	€173.28	$230.10
				**Physician Visits and Hospitalizations**				
Papanicolaou 2005 [35]	Germany	Payer	1 yr	Office visits	2004	€45	€49.43	$65.64
				Surgery		€95	€104.35	$138.57
	Spain			Office visits		€168	€195.20	$259.20
				Surgery		€103	€119.68	$158.92
	UK/Ireland			Office visits		€95	€110.99	$147.38
				Surgery		€40	€46.73	$62.05
Tediosi 2000 [36]	Italy	Payer	1 yr	Hospitalizations	1997	L19.814	L26.09	$34.64
Thom *et al*. 2005 [38]	US	Payer	1 y	Hospitalizations, physician visits, ED visits	1998	$7,702 USD	$12,357.43 USD	$12,357.43
				**Accommodations**				
Irwin *et al*. 2009 [36]	Canada	Payer	1 yr	Nursing home	2005	€385	€419.15	$556.59
	Germany			Nursing home		€1,038	€1,130.10	$1,500.65
	Italy			Nursing home		€1,580	€1,720.19	$2,284.22
	Spain			Nursing home		€30	€32.66	$43.37
	Sweden			Nursing home		€562	€611.87	$812.50
	UK			Nursing home		€381	€414.81	$550.82
Prasopsanti *et al*. 2007 [41]	Thailand	Patient	1 yr	Transportation	2002	$198.12	$238.11	$238.11

^*^ Costs were not extrapolated for this study because costs from the study were costs for sub-acute care, not routine care.

*UI* = urinary incontinence; *LTC* = long-term care facility; *UTI* = urinary tract infection; *ED* = emergency department.

The one study that assessed the economic impact of NDO suggests that direct costs of conservative care per patient in European countries may be higher in patients with UI due to NDO compared with patients with UI in general or due to other causes [15]. In this study, which evaluated medical resource use before and after treatment with botulinum toxin A in patients with NDO, the direct costs of NDO (reported from a third-party payer perspective prior to treatment) included urinary tract infection (UTI) pharmacotherapy and incontinence aids (pads, urinary reservoirs).Annual per-patient pharmacotherapy costs for UTI were calculated to be $230.10, whereas incontinence aids were calculated at $2.97 per patient per day, which could be as much as $1,085.68 per year if extrapolated to annual cost in US dollars. The estimated annual payer costs per patient for pad usage was $727.02, which is much higher compared with pad costs reported in other European countries (Table 2) [15].

Irwin *et al*. [36] reported costs from the payer perspective in various countries reporting annual direct costs for OAB and urgency UI. Associated costs included physician office visits, hospital outpatient services, surgeries, emergency room services, nursing home care, conservative care and devices, pad usage, and medications for symptoms of OAB, including UI. Annual payer costs per patient for incontinence pad usage ranged from $72.97 (in the UK) to $152.24 (in Spain). The study also reported per-patient costs for UTIs, skin infections, fractures, and nursing home admissions associated specifically with urgency UI. The annual costs per patient for urgency UI–related comorbidities ranged from $10.12 (in Spain) to $50.61 (in Sweden), whereas the annual urgency UI–related nursing home costs per patient ranged from $43.37 (in Spain) to $2,284.22 (in Italy).

When acute hospitalization and long-term care costs are added, the annual cost estimates increase further for patients with UI. A study evaluating the cost of UI in 54 subacute medical facilities in Australia and New Zealand found that stroke patients with high FIM motor scores were more expensive to treat if they were incontinent [34]. Another study conducted among a consecutive series of patients admitted to one of two sub-acute care facilities in Australia who had UI found that the total median daily cost of caring for UI among patients with UI of neurological origin was $19.76 [33]. One study in the United States reported that a payer spends as much as $12,357.43 per female UI patient annually for hospitalizations, physician office visits, outpatient services, and emergency department services, with hospitalization accounting for a majority of the costs [38]. Two other studies assessed the provider costs associated with UI in patients in long-term care facilities in the United States. Franz *et al*. [39] reported that 6-month nursing staff costs were $2,416.94 per UI patient. Six-month incontinence management costs per patient were also substantial, with toileting (time spent to toilet a patient by a nursing assistant) accounting for the highest cost component (Table 2). Shih *et al*. [40] analyzed the annual incremental cost of labor associated with UI in the long-term facilities alone and found that the annual cost of labor per UI patient to be $6,741.87.

Few studies reported per-patient costs for UI from the societal or patient perspective. A study of costs associated with OAB, including UI, in Thailand reported that the weekly cost for pad usage was $4.45 for a patient with UI, which, if extrapolated to annual cost, could be as much as $232.20 per year [41]. In the United States, the weekly cost for personal care for a patient with UI (includes pads, paper towels, laundry, and dry cleaning) was $7.90, which, if extrapolated to annual costs, could be as high as $412.20 per year [42].

## Discussion

To our knowledge this is the first study to characterize the HRQoL and economic burden of UI in patients with different underlying neurologic conditions. We found that UI in individuals with MS, SCI, Parkinson’s disease, and stroke has a substantial negative impact on patients’ HRQoL. Physical, mental and psychological impairments were consistently observed, with patients reporting detriments in physical function, emotional well being, and social relationships. UI also had a negative effect on the sexual lives of patients with neurologic conditions, and may adversely affect long-term outcomes. Not surprisingly, we also found that incontinent individuals with underlying neurologic conditions also typically report worse HRQoL compared with their continent counterparts and the general population.

Three studies suggested that UI has little or no impact on the HRQoL of patients with MS or spina bifida [16,24,32]. Two of these studies used the SF-36, which suggests that a general HRQoL instrument may not always be sensitive to capturing the impact of UI in populations that already have very debilitating conditions. Another study which used the KHQ reported that incontinent MS patients did not consider their incontinence to be severe [18]. This could be attributed to an accommodation that occurs in patients with chronic conditions such as MS. These patients often need to cope with other symptoms of MS that may be more debilitating when compared with UI. These findings point to the need for additional research, using disease-specific instruments to further understand the impact of UI on the HRQoL of patients with MS or spina bifida.

Evaluating the economic impact of a disease or disease intervention is becoming increasingly important and is often one of the factors considered in determining access to treatment for patients. However, very little economic information regarding UI due to NDO or in patients with MS, SCI, Parkinson’s disease, stroke, or spina bifida was available, pointing to a need for further research to assess the economic burden of UI in these specific neurologic populations. Nevertheless, the results do suggest that the economic burden of UI due to NDO is substantial for patients, providers and payers, including direct medical costs, as well as costs for conservative care, physician/hospital/emergency room services, medication and complications.

Furthermore, existing studies that describe the economic burden of UI have mixed populations, where costs for UI are lumped with that for OAB as a whole. We were primarily interested in the economic impact of UI on an individual level rather than on a population level, recognizing that the general OAB population is substantially larger and distinct from patients with underlying neurologic conditions who experience bladder dysfunction. The studies that provided per-patient annual direct costs for UI from a payer perspective were mostly conducted in European countries, with annual costs ranging from $488 to $949 per patient [36]. Meanwhile, studies in the United States indicate that payers spend as much as $12,353 annually per UI patient for hospitalizations, physician office visits, outpatient services, and emergency department services [38]. Assuming that 72% (n = 288,000) of the approximately 400,000 MS patients in the United States have UI [43,44], the estimated direct costs of UI attributable to MS for payers can be as much as $3.5 billion annually. This represents substantial incremental costs to an already costly condition, which has an estimated total annual cost of $10 billion (2010 US$ inflated from $6.8 billion 1994US$) [45]. Further research on the economic burden of UI in these patients with chronic, neurologic diseases may be necessary to ensure access to novel therapies that could treat this condition.

We found a great deal of heterogeneity in the studies we identified with respect to study design, patient characteristics, and the types of outcome measures used to characterize the humanistic and economic burden of UI. This was true both within and across the neurologic conditions of interest. For example, studies examining the impact of UI on the HRQoL in MS patients often used different instruments than studies that assessed the impact of UI in SCI patients. Similarly, the economic studies that we identified and reviewed applied different cost components (e.g., medication costs, diagnostic costs, office visit costs, labor costs) when quantifying the economic burden of UI. As such, the ability to compare the humanistic and economic impact of UI across neurologic conditions and across countries was limited. Our results thus suggest the need for the medical community to reach a consensus on which tool(s) should be used to assess HRQoL in patients with neurologic diseases, so that researchers have a standard guideline to follow when designing clinical trials. Use of the same tool across studies will also facilitate future comparisons.

Our search was limited by the inconsistent terminology used across the literature to describe this subset of patients with UI due to NDO. As such, studies that used terms different from ours may not have been generated in our search. No relevant studies were found in patients with UI due to urodynamically confirmed NDO, which is not surprising given that urodynamic evaluations are not routinely done in patients with urinary symptoms and that the terminology surrounding lower urinary tract function has constantly evolved in the last decade [1]. Furthermore, because the studies we reviewed to assess the economic burden of UI were largely conducted in the general UI population and not in patients with MS, SCI, Parkinson’s disease, stroke, or spina bifida, the results presented here are exploratory and, with the exception of Wefer *et al*. [15], not necessarily reflective of costs associated with treatment of UI due to NDO. Studies evaluating the economic impact in this subset of patients are needed.

Important goals of therapy for neurogenic lower urinary tract dysfunction should focus on improving patient HRQoL by decreasing the most bothersome symptoms as much as possible [46,47]. Future research is still needed to clearly assess the burden of UI due to NDO in patients with MS, SCI, Parkinson’s disease, stroke, spina bifida, and other neurologic conditions in which UI is an associated symptom of the disease. These studies should be carefully designed to include urodynamic observations to confirm the presence of NDO, consistent and validated HRQoL instruments, and clearly defined cost components.

## Conclusion

Incontinent patients with MS, SCI, Parkinson’s disease, stroke, or spina bifida have impaired HRQoL. In particular, detriments in physical function, emotional well being, and social relationships were generally noted across studies. HRQoL of incontinent patients with neurologic conditions is also significantly worse compared with patients with UI in general or patients with the same underlying neurologic condition without UI. Our results also suggest that the economic burden of UI due to NDO is substantial, and that there is a need for UI treatments that improve HRQoL and alleviate the economic burden of this condition.

## Abbreviations

ACS: Activity Card Sort; ADL: Activities of daily living; AUA: American Urological Association; CI: Confidence interval; CIC: Clean intermittent cauterization; FAM: Functional Assessment Measure; FIM: Functional Independence Measure; HRQoL: Health-related quality of life; HUI3: Health Utilities Index Mark; IIQ-7: Incontinence Impact Questionnaire; I-QOL: Incontinence Quality of Life questionnaire; KHQ: Kings Health Questionnaire; MS: Multiple sclerosis; MCS: Mental component summary; NDO: Neurogenic detrusor overactivity; OAB: Overactive bladder; OAB-Q SF: Overactive Bladder Questionnaire Short Form; PCS: Physical component summary; RNL: Reintegration to Normal Living Index; SCI: Spinal cord injury; SIP: Sickness Impact Profile; SF-12: Medical Outcomes Study-12-Item Short Form Survey; SF-36: Medical Outcomes Study 36-Item Short Form Health Survey; UDI-6: Urogenital Distress Inventory; UI: Urinary incontinence; UTI: Urinary tract infection; VSP: Vecu et santé percu

## Competing interests

This study was funded by Allergan, Inc. CI and KB declare that they are employees of Covance Market Access Services, Inc.; KK and DG declare that they are employees of Allergan, Inc.; Dr Chancellor declares that he is an advisor and consultant for Allergan, Inc. and has conducted studies funded by Allergan, Inc., and Dr Carr declares that she is a consultant for Allergan, Pfizer, Astellas, Triton, Watson, Cook Myosite, Gynecare and Ferring and has conducted studies funded by Cook Myosite, Allergan, Astellas and Pfizer.

## Authors’ contributions

CT, KK, DG, KB, MC, and LC participated in the design of the study, including development of the literature search protocol. CT conducted the systematic literature review. DG and KK conceived of the study, and participated in its design and coordination. All authors helped to draft the manuscript, and all authors read and approved the final manuscript.

## Supplementary Material

Additional file 1: Table S1 Studies on the Impact of UI on the HRQoL of Patients with Underlying Neurological Conditions and Key Findings. Click here for file
