# Supplementary material for: Health-related quality of life and economic impact of urinary incontinence due to detrusor overactivity associated with a neurologic condition: a systematic review
Source: Health Qual Life Outcomes. 2013 Jan 31;11:13. doi: 10.1186/1477-7525-11-13 (PMC3606444; doi:10.1186/1477-7525-11-13)
Supplement: Additional file 1: Table S1 — Studies on the Impact of UI on the HRQoL of Patients with Underlying Neurological Conditions and Key Findings. [file 1477-7525-11-13-S1.doc]

**Table S1** **Studies on the Impact of UI on the HRQoL of Patients with Underlying Neurological Conditions and Key Findings**

| **Citation**; **CEBM Evidence Grade**[9] | **Study Design and Outcome Measures** | **Country** | **Populations** | **Key Findings** |
| --- | --- | --- | --- | --- |
| Edwards *et al*. 2006 [27]; 3 | Study Design: Telephone interviews | US | Stroke patients | • UI once per month or more is associated with diminished QoL and activity participation  • Incontinent patients had significantly lower scores (poorer functioning and HRQoL) compared with continent patients (p < 0.001)  • Poor life satisfaction (as measured by RNL) was associated with ADL impairment, cognitive disability, low SF-12 PCS and MCS scores, and incontinence  • Poor outcome was independent of stroke severity  • Low scores on the SF-12 physical and mental health scales were associated with diminished life satisfaction  • Poor performance on the FIM cognitive scale was the single strongest predictor of low life satisfaction  After accounting for the contributions of all other factors, UI was independently associated with poor life satisfaction (OR 4.15, 95% CI: 1.34-12.09) |
|  |  |  | • N = 361, mean age 65.52 ± 15.24 yr  • 53% (n = 201) were female  • Continent (n = 301)  Mean age 64.56 ± 15.18 yr  54% (n = 165) were female  • Incontinent (n = 59)  Mean age 70.14 ± 14.83 yr 60% (n = 36) were female |  |
|  | Outcome Measures:  • NIHS  • FIM  • FAM  • SIP  • SF-12  • Activity Card Sort Checklist  • RNL |  |  |  |
| Forbes *et al*. 2006 [16]; 3 | Study Design: Cross-sectional postal survey | England | Patients with MS | • 74% of the sample experienced UI  • UI was not found to have an effect on SF-36 scores  • SF-36 scores were most compromised in patients experiencing multiple problems |
|  |  |  | • N = 929, mean age 48 ± 11.4 yr  • 69% were female  • 74% with UI |  |
|  | Outcome Measures:  • MSIS-29  • SF-36 |  |  |  |
| Goris *et al*. 2010 [17]; 3 | Study Design: Descriptive survey study | Turkey | Women with MS | • 45% had UI, 33.3% had mixed type and 48.2% had incontinence every day; 44.4% had incontinence as much as to wet underwear and used pads daily (55.6%); 63.0% did not visit the doctor  • UI was higher in women aged >45 y, married with children, and diagnosed for 6 yr (p < 0.05)  • 28.3% had sexual dysfunction  • Average QoL scores among women with MS for physical functioning: UI = 42.87 ± 33.61; without UI = 62.42 ± 24.78 (p < 0.05) |
|  |  |  | • N = 60  • Mean age 35.93 ± 9.35 yr  • 45% with UI |  |
|  | Outcome Measures:  • ASEX  • SF-36 |  |  |  |
| Hollingworth *et al*. 2010 [23]; 2 | Study Design: Assessment preference-based outcomes from a randomized controlled trial | US | Patients with UI due to SCI (n = 53) or MS (n = 6) | • Baseline data: Mean (SD) overall I-QOL score, n = 56: 43.7 (20.8); avoidance and limiting behavior, n = 56: 48.7 (23.1); psychosocial impacts, n = 56: 45.2 (23.8); social embarrassment, n = 56: 33.0 (24.0)  • Mean (SD) SF-36 dimension scores: physical function, n = 48: 31.6 (25.1); role-physical, n = 54: 52.8 (44.4); bodily pain, n = 56: 57.7 (27.4); general health, n = 56: 51.7 (18.6); energy/vitality, n = 55: 51.3 (18.9); social function, n = 56: 65.4 (26.0); role-emotional, n = 54: 62.3 (43.9); mental health, n = 55: 62.2 (17.5) |
|  |  |  | • Mean age 41.2 ± 13.3 yr  • 40% were female |  |
|  | Outcome Measures: |  |  |  |
|  | • I-QOL  • SF-36  • SF-6D |  |  |  |
| Iacovelli *et al*. 2010 [25]; 3 | Study Design: Prospective cohort study | Italy | Patients with Parkinson’s disease | • OAB-Q had significantly higher scores in patients with Parkinson’s disease vs. healthy subjects (18.71 ± 8.9 vs. 12.37 ± 5.1; p = 0.0001), indicating that patients with Parkinson’s disease and urinary symptoms were significantly more bothered compared with healthy controls |
|  | Outcome Measures: |  | • Males (n = 168): age 69.2 ± 9.1 yr  • Females (n = 134): age 69.72 ± 9.2 yr |  |
|  | • OAB-q  • Unified Parkinson’s Disease Rating Scale Part III  Hoehn-Yahr staging |  |  |  |
|  |  |  |  | • OAB-Q values were significantly with age and UPDRS-III scores: (R2 = 0.139; age p = 0.006, UPDRS-III p = 0.002; disease duration p = 0.76; H-Y staging p = 0.16) |
| Khan *et al*. 2009 [18]; 2 | Study Design: Cross-sectional study | Australia | Patients with MS | • Of 73 patients, two thirds were bothered by urinary frequency, half reported UI, and 14% bowel incontinence  • Urinary problems impacted emotional health (31%), ability to perform household chores (22%), and physical recreation (28%), with detrimental impact on QoL  • A significant relationship between symptoms, level of urogenital distress (rho = 0.74, p < 0.001) and impact of incontinence (rho = 0.68, p < 0.001) was seen  • The single item of AUA Symptom Index assessing impact of bladder symptoms on QoL correlated significantly with all other bladder scales (rho = 0.60-0.74) |
|  |  |  | • N = 73, mean age 50 yr  73% were female |  |
|  | Outcome Measures:  • NDS  • AUA Symptom Index  • IIQ7  • UDI6  • WFIS  • MSIS 29  • GHQ28 |  |  |  |
| Kolominski-Rabas *et al*. 2003 [29]; 2 | Study Design: Prospective population-based stroke registry | Germany | Stroke patients  • N = 752  • UI at day 7 post stroke (n = 172)Mean age 68.1 (69) yr  51% were female  • UI at 12 mo post stroke (n = 130)  Mean age 74.7 (77) yr  59% were female | • At 12 mo, 45% of incontinent patients were institutionalized compared with 5% without UI  • Of 130 patients with UI at 12 mo, 7% were fully incontinent, 28% partially incontinent, and 65% continent prior to experiencing a stroke |
|  | Outcome Measures:  • Barthel Index  • Rate of institutionalization  • Mortality |  |  |  |
| Lemelle *et al*. 2006 [32]; 3 | Study Design: Clinical interviews and retrospective chart review study | France | Patients with SCI and myelomeningocele  • Adults (aged ≥18 yr): n = 300Mean age: 27.07 ± 6.89 (range 18–51) yr  • Adolescents (aged 10–17 yr): n = 160  Mean age 14.44 ± 1.84 yr  • Sex ratio: Adults 1.13: adolescents 1.19 | • No strong relationship between incontinence and HRQoL was found  • Comparison between HRQoL scores in UI and urinary continent adults and adolescents did not show significant difference except for ‘bodily pain’ dimension, in which higher score was noticed in continent group (p = 0.05) |
|  | Outcome Measures: |  |  |  |
|  | • SF-36 (adults)  • VSP (adolescents) |  |  |  |
| Liu *et al*. 2010 [21]; 3 | Study Design: Cross-sectional study | UK | Patients with SCI | • Patients without incontinence had the best scores (highest QoL) in all SF-36 domains, with significant differences in mental health (p = 0.041) and MCS (p =0.001)  • Patients without incontinence had the best results (highest QoL as measured by the KHQ) compared with patients with daily incontinence, which had the worst results (lowest QoL)  • Significant hierarchical differences were observed in incontinence impact, social limitations, emotions, and severity of bladder problem  • Patients without incontinence also had the best results (lowest scores), in contrast to persons with daily incontinence who had the worst results (highest scores) |
|  |  |  | • N = 142  • Mean age 45.2 ± 14.6 yr  • 26.1% were female |  |
|  | Outcome Measures: |  |  |  |
|  | • SF-36  KHQ |  |  |  |
| Oh *et al*. 2005 [22]; 2 | Study Design: Prospective trial | South Korea | Patients with SCI using CIC due to neurogenic bladder secondary to SCI  • Patient group: n = 132  • Matched controls: n = 150  • Mean age 41.8 ± 1.4 yr  • 38.86% were female | • No significant differences were found between men and women in SF-36 scores  • Incontinent SCI patients aged ≥50 y had significantly lower scores for energy and vitality than those <50 yr (p = 0.003)  • Incontinent SCI patients’ SF-36 scores were significantly lower than those of general population  • When incontinent SCI patients and controls were divided into two groups according to gender and age, SF-36 scores of SCI patients were significantly lower than controls across both gender and age groups |
|  | Outcome Measures: |  |  |  |
|  | SF-36 |  |  |  |
| Patel *et al*. 2001 [31]; 3 | Study Design: Prospective, observational study | UK | Stroke patients | • Rankin Scale: at 2 yr, handicap was also worse in Group 1 vs. Group 2 (p = 0.013)  • At 2 yr, compared with continent group, incontinent group had higher case-fatality rates (67% vs. 20%; p < 0.001), higher institutionalization rates (39% vs. 16%; p = 0.007), and greater disability (Barthel [0–9]: 39% vs. 5%; p < 0.001; Frenchay [0–15]: 75% vs. 37%; p = 0.001)  • Death or disability at 2 yr was worse in patients with initial incontinence (OR 4.43; 95% CI: 1.76-11.2) |
|  |  |  | • n = 127 regained continence at 3 mo  • n = 60 remained incontinent at 3 mo  • Patients who regained continence:  Age: 67.0 ± ±13.30 yr  55.9% (n=71) were female  • Patients who remained incontinent:  Age: 73.1 ± 13.23 yr  47.5% (n = 38) were female |  |
|  | Outcome Measures:  • Barthel Index  • Frenchay Activity Index  • Rate of institutionalization |  |  |  |
| Patel *et al*. 2001 [30]; 3 | Study Design: Prospective, population-based 2-y stroke registry study | UK | N = 235 | • Ages ≥75 yr (OR 0.38; 95% CI: 0.17-0.83) was associated with poor recovery from incontinence  • At 3 mo, the incontinent group had greater institutionalization rates (n = 27 [34%] vs. 9 [7%], p < 0.001) and worse disability, measured with BI and FAI (BI: p < 0.001, FAI: p = 0.002) |
|  |  |  | • Group 1: n = 95 (40% of total)  Mean age (SD) 73.33 (14.02) yr  57% were female  • Group 2: n = 140  Mean age (SD) 69.68 (13.96) yr  46% were female |  |
|  | Outcome Measures:  • Barthel Index  • Frenchay Activities Index  • Rankin Scale  Rate of institutionalization |  |  |  |
| Pohar *et al*. 2009 [26]; 3 | Study Design: Survey study | Canada | • Patients with Parkinson’s disease:  n = 261  Mean age 68.9 yr 44.1% were female  • Control/general population: n = xxxx, mean age 44.8 yr  51.0% were female | • Overall HRQoL scores were lower in persons with Parkinson’s disease who also reported having arthritis, back problems, UI, or cataracts than in persons with Parkinson’s disease who did not have these conditions  • Only UI (OR −0.22, 95% CI: –0.40 to −0.05) and arthritis (−0.17, 95% CI: –0.03 to −0.04) were statistically significant (p < 0.05)  • Clinically important and statistically significant differences were reported in the following HUI3 domains: ambulation OR −0.22, 95% CI: –0.42 to −0.02 (p < 0.05) and emotion OR −0.28, 95% CI: –0.43 to −0.12 (p < 0.05) between respondents with Parkinson’s disease with UI vs. without UI |
|  | Outcome Measures:  • HU13 |  |  |  |
| Tibaek *et al*. 2009 [28]; 3 | Study Design: Cross-sectional clinical survey | Denmark | Stroke patients  • N = 407  • Mean age 67 ± 12 yr  • 46% were female | • One of the most frequent symptoms among stroke patients was urinary urgency (70%)  • Low BI and reduced mobility velocity increase the risk of prevalence of incontinence symptoms: OR 2.08, 95% CI: 1.09-3.98 (p = 0.03) and OR 1.87, 95% CI: 1.00-3.50 (p = 0.05), respectively  • Low BI and reduced mobility distance had a significant effect on severity in the incontinence symptom group (p = 0.03 and p = 0.04, respectively)  • Low BI significantly increased the prevalence of bother in the incontinence symptom group: OR 2.12, 95% CI: 1.10-4.08 (p = 0.02)  • Overall, UI symptom groups showed that BI was significantly associated with the prevalence of symptoms, severity. and prevalence of LUTS |
|  | Outcome Measures:  • Barthel Index  • DAN-PSS-1 |  |  |  |
| Quarto *et al*. 2007 [19]; 2 | Study Design: | Italy | Women with MS and urinary symptoms  • OAB +MS: 31.7% with urge incontinence  • OAB (control): 34% with urge incontinence | • Urinary symptoms influence patients with MS much more than patients without MS  • A statistically significant difference was observed in the following SF-36 domains, indicating that incontinent MS patients having worse HRQoL *vs*. continent patients, respectively: general health perception, 56.3 vs. 33.3 (p = 0.02); role limitation, 50 vs. 36.1 (p = 0.03); physical limitation, 56.3 vs. 33.3 (p = 0.02); social limitation, 45.8 vs. 18.5 (p = 0.002); and urinary symptoms, 17 vs. 14 (p = 0.05) |
|  | Prospective observational study |  |  |  |
|  | Outcome Measures:  • EDSS  • KHQ |  |  |  |
| Valtonen *et al*. 2006 [24]; 3 | Study Design: Cross-sectional survey study | Sweden | Patients with traumatic SCI and meningomyelocele  • Traumatic SCI (n = 190):  Mean age (range) 46.6 (21.8-74.2) yr  24.2% were female  • Meningomyelocele (n = 41):  Mean age (range) = 31.1 (19.6-50.5) yr  43.9% were female | • In the group with traumatic SCI, median of self-assessed inconvenience caused by UI was 5 for men and 3 for women  • Those reporting more inconvenience from UI were more dissatisfied with their sexual life than those with lower scores for inconvenience (association significant for men and not for women): spearman rho =−0.19 (p = 0.021) and −0.18 (p = 0.236), respectively  • In the group with meningomyelocele, median of self-assessed inconvenience caused by UI was 4 for men and 4 for women  • The self-assessed inconvenience caused by UI did not affect sexual satisfaction in the persons with meningomyelocele |
|  | Outcome Measures:  • Satisfaction with sexual life questionnaire, including a question about inconvenience caused by UI (i.e., “How would you rate your inconvenience caused by urinary incontinence?) |  |  |  |

Abbreviations: *ADL* = activities of daily living; *ASEX*= Arizona Sexual Experiences Scale; *AUA* = American Urological Association; *BI* = Barthel Index; *CEBM* = Oxford Centre for Evidence-Based Medicine; *CI* = Confidence Interval; *CIC* = clean intermittent catheterization; *DAN*-*PSS*-*1* = Danish Prostatic Symptom Score Questionnaire; *EDSS* = Expanded Disability Status Scale; *FAI* = Frenchay Activities Index; *FIM* = Functional Independence Measure; *FAM* = Functional Assessment Measure; *GHQ28* = General Health Questionnaire 28; *HUI3* = Health Utilities Index; *H*-*Y* = Hoehn and Yahr Rating Scale; *IIQ7* = Incontinence Impact Questionnaire; *I*-*QOL* = Incontinence Quality of Life; *KHQ* = Kings Health Questionnaire; *LUTS* = lower urinary tract symptoms; *MCS* = mental component summary; *MS* = multiple sclerosis; *MSIS 29* = Multiple Sclerosis Impact Scale-29; *NDS* = Neurological Disability Scale; *NIHS* = National Institutes of Health Stroke Scale; *OAB*-*Q* = Overactive Bladder questionnaire; *OR* = odds ratio; *PCS* = physical component summary; *RNL* = Reintegration to Normal Living Index; *SCI* = spinal cord injury; *SD* = standard deviation; *SF*-*12* = Medical Health Outcomes Survey Short Form-12; *SF*-*36* = Medical Health Outcomes Survey Short Form-36; *SF*-*6D* = preference-based measure of health from the SF-36; *SIP* = Stroke-adapted Sickness Impact Profile; *UDI6* = Urogenital distress Inventory; *UPDRS III* = Unified Parkinson’s Disease Rating Scale; *VSP* = Vecu et sante´ percu; *WFIS* = Wexner Faecal Incontinence Score; *QoL* = quality of life; *HRQoL* = health-related quality of life; *UI* = urinary incontinence;

Abbreviations: *CEBM* = Oxford Centre for Evidence-Based Medicine; *UI* = urinary incontinence; *NIHS* = National Institutes of Health Stroke Scale; *FIM* = Functional Independence Measure; *FAM* = Functional Assessment Measure; *SIP* = Stroke-adapted Sickness Impact Profile; *SF*-*12* = Medical Health Outcomes Survey Short Form-12; *RNL* = Reintegration to Normal Living Index; *MS* = multiple sclerosis; *MSIS 29* = Multiple Sclerosis Impact Scale-29; *SF*-*36* = Medical Health Outcomes Survey Short Form-36; *ASEX*= Arizona Sexual Experiences Scale; *NDS* = Neurological Disability Scale; *AUA* = American Urological Association; *IIQ7* = Incontinence Impact Questionnaire; *UDI6* = Urogenital distress Inventory; *WFIS* = Wexner Faecal Incontinence Score; *GHQ28* = General Health Questionnaire 28; SCI = spinal cord injury; *I*-*QOL* = Incontinence Quality of Life; *SF*-*6D* = preference-based measure of health from the SF-36; *OAB*-*Q* = Overactive Bladder questionnaire; *VSP* = Vecu et sante´ percu; *KHQ* = Kings Health Questionnaire; *CIC* = clean intermittent cauterization; *SD* = standard deviation; *HUI3* = Health Utilities Index; *DAN*-*PSS*-*1* = Danish Prostatic Symptom Score Questionnaire; *EDSS* = Expanded Disability Status Scale
